# The Role of Lipids in *Legionella*-Host Interaction

**DOI:** 10.3390/ijms22031487

**Published:** 2021-02-02

**Authors:** Bozena Kowalczyk, Elzbieta Chmiel, Marta Palusinska-Szysz

**Affiliations:** Department of Genetics and Microbiology, Institute of Biological Sciences, Faculty of Biology and Biotechnology, Maria Curie-Sklodowska University, Akademicka St. 19, 20-033 Lublin, Poland; b.kowalczyk746@wp.pl (B.K.); ela.wisniewska87@gmail.com (E.C.)

**Keywords:** *Legionella*, phosphatidylcholine, lipopolysaccharide

## Abstract

*Legionella* are Gram-stain-negative rods associated with water environments: either natural or man-made systems. The inhalation of aerosols containing *Legionella* bacteria leads to the development of a severe pneumonia termed Legionnaires’ disease. To establish an infection, these bacteria adapt to growth in the hostile environment of the host through the unusual structures of macromolecules that build the cell surface. The outer membrane of the cell envelope is a lipid bilayer with an asymmetric composition mostly of phospholipids in the inner leaflet and lipopolysaccharides (LPS) in the outer leaflet. The major membrane-forming phospholipid of *Legionella* spp. is phosphatidylcholine (PC)—a typical eukaryotic glycerophospholipid. PC synthesis in *Legionella* cells occurs via two independent pathways: the *N*-methylation (Pmt) pathway and the Pcs pathway. The utilisation of exogenous choline by *Legionella* spp. leads to changes in the composition of lipids and proteins, which influences the physicochemical properties of the cell surface. This phenotypic plasticity of the *Legionella* cell envelope determines the mode of interaction with the macrophages, which results in a decrease in the production of proinflammatory cytokines and modulates the interaction with antimicrobial peptides and proteins. The surface-exposed O-chain of *Legionella pneumophila* sg1 LPS consisting of a homopolymer of 5-acetamidino-7-acetamido-8-O-acetyl-3,5,7,9-tetradeoxy-l-glycero-d-galacto-non-2-ulosonic acid is probably the first component in contact with the host cell that anchors the bacteria in the host membrane. Unusual in terms of the structure and function of individual LPS regions, it makes an important contribution to the antigenicity and pathogenicity of *Legionella* bacteria.

## 1. Introduction

**Figure 1:**
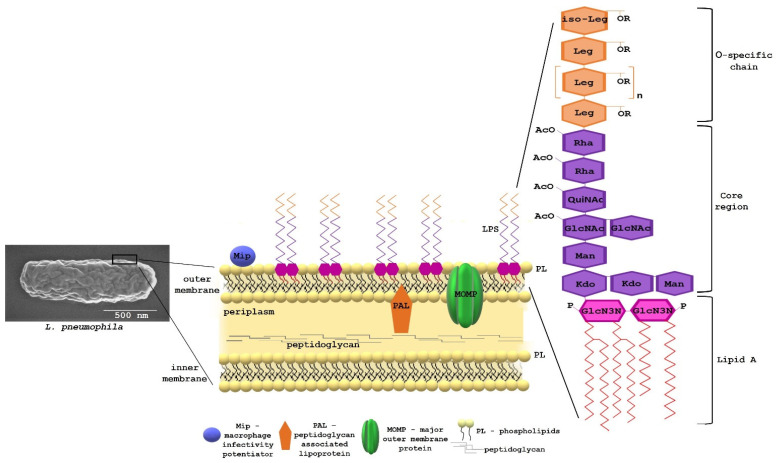
Scanning electron microscopy image of *Legionella pneumophila*, and model of the *L. pneumophila* cell envelope. Structure of lipopolysaccharide (LPS) (modified from [16]). Leg., legionaminic acid; Rha, rhamnose; OAc, O-acetyl; QuiNAc, acetylquinovosamine; GlcNAc, acetylglucosamine; Man, mannose; Kdo, 3-deoxy-d-manno-oct-2-ulosonic acid; P, phosphate; and GlcN3N, 2,3 diamino-2,3-dideoxy-d-glucose.

Bacteria from the family *Legionellaceae* are Gram-negative bacilli, which are part of the natural microflora of aquatic and soil environments, as well as anthropogenic ecosystems. From the microbiological point of view, the ecology of *Legionella* bacilli is highly complex. *Legionella* bacteria survive in a temperature range between 5 °C and 65 °C and at pH 7–9; however, due to their specific nutritional requirements and a narrow range of environmental conditions necessary for their growth, they are unable to compete with other bacteria [1]. The bacteria require specific physicochemical conditions for development, which can only be fulfilled inside the eukaryotic host. Protozoa, commonly found in natural ecosystems, are an important link in the food chain and exert a significant impact on bacterial populations. However, not all bacteria are ingested, killed, and digested by protozoa. The outcome of the *Legionella*–protozoa interaction is associated with the host species. In some cases, *Legionella* resists digestion and kills the host, and in others, the protist digests the bacterium [2]. A total of 61 different *Legionella* species and more than 70 serogroups have been identified [3]. An in-silico analysis of the *Legionella pneumophila* genome identified several genomic islands indispensable for bacterial growth within protozoa, which differ among the various amoeba species [4]. Free-living amoebae from the genus *Acanthamoeba*, *Naegleria*, and *Vermamoeba* (*Hartmanella*) and ciliates from the genus *Tetrahymena* provide an intracellular niche in which *Legionella* can proliferate [5,6]. The intracellular growth of the bacteria has been associated with the enhanced environmental survival, virulence, and antibiotic resistance of the bacteria [7,8]. *L. pneumophila*, i.e., the most pathogenic species of this family, use many molecular and cellular aspects of intracellular proliferation inside protozoa in patho-adaptation to human cells [9]. *Legionella* enter the human organism from the natural environment via contaminated aerosols generated by cooling towers, large air-conditioning systems, fountains, showers, groundwater used for sprinkler irrigation, and similar sources [10,11]. This mode of bacterial spread is regarded as the major infection route, although bacterial transmission from person to person has been reported as well [12]. In the human organism, the bacteria cause respiratory infections with varying severity: from a flu-like infection called Pontiac fever, which does not require specialised treatment, to an acute, multi-lobar pneumonia called Legionnaires’ disease, which may result in death [13]. The bacteria proliferate robustly in lung alveolar macrophages, leading to tissue damage and the subsequent development of the disease. The macrophage resistance of *Legionella* spp. is a prerequisite for their virulence. Bacterial survival in contact with eukaryotic cells—in particular, those predisposed for killing bacteria—depends on many determinants of adaptation to an environment that is extremely hostile but rich in nutrients. The bacteria overcome the killing mechanisms of phagocytes by means of specialised protein translocation systems: type II Lsp and type IVB Dot/Icm. The type II system controls the secretion of enzymes (lipases, protease, and RNase), thus promoting growth, intracellular replication, and virulence [14]. The Dot/Icm transporter delivers substrates that modulate multiple host cell processes, resulting in the biogenesis of the *L. pneumophila*-containing vacuole (LCV) permissive for intracellular bacterial replication. *L. pneumophila* achieve host infection by exporting approximately 300 substrates of the Dot/Icm system across one or two cell membranes to the site of action [15]. The precise delivery of the effectors to the host cell in a timely manner and space is possible thanks to an efficiently functioning bacterial cell envelope. The cell envelope of *L. pneumophila* is typical for Gram-negative bacteria and consists of two distinct membranes, the inner (IM) and outer membrane (OM), separated by the periplasm. The periplasm contains a relatively thin layer of strongly crosslinked peptidoglycan and various proteins. Peptidoglycan is composed of muramic acid, glucosamine, glutamic acid, alanine, and meso-diaminopimelic acid in a molar ratio of 0.8:0.8:1.1:1.7:1 [16]. The OM is asymmetric, with an inner leaflet mostly composed of phospholipids and an outer leaflet mostly comprising lipopolysaccharides (LPS). This asymmetry is critical for maintaining the OM permeability barrier. LPS consists of three regions: O-antigen, core, and lipid A. The lipid A region anchors LPS molecules to the outer membrane through hydrophobic interactions with the acyl chains of the phospholipids (PLs) constituting the inner layer of this membrane [17]. In addition to proteins, which have a fundamental importance for various aspects of cell physiology, including the infection of a host organism, lipids are involved in the highly specific interactions with the host cell. Three types of lipid-containing molecules are present in the cell envelope of *L. pneumophila*: phospholipids (PL), lipopolysaccharide (LPS), and lipoproteins (Figure 1).

## 2. Characteristics of *Legionella* Phospholipids

Most bacterial membranes are formed by amphiphilic lipids, mainly glycerophospholipids. Phospholipids are composed of a variable hydrophilic head group, two fatty acids esterified in the *sn-1* and *sn-2* positions of the glycerol moiety, which form a hydrophobic tail, and a phosphate group in the *sn-3* position. Phospholipids fulfil a passive role as building blocks of the lipid bilayer and stimulate cell functions, e.g., targeted protein transport, DNA replication, or signal transduction. The composition and distribution of phospholipids in the membrane, their ability to undergo hydrolysis, and the modifications of the phospholipid “head-groups” constitute a specific “molecular language” in the interactions with other cell molecules and the environment [18].

### 2.1. Phospholipids of Legionella spp.

The most typical bacterial phospholipids include phosphatidylethanolamine (PE), phosphatidylglycerol (PG), and cardiolipin (CL). Zwitterionic PE is found in many groups of Gram-negative bacteria (e.g., *Enterobacteriaceae*) and in several Gram-positive bacteria, while anionic PG and CL are common in Gram-positive bacteria such as enterococci, streptococci, and staphylococci [19,20].

**Figure 2:**
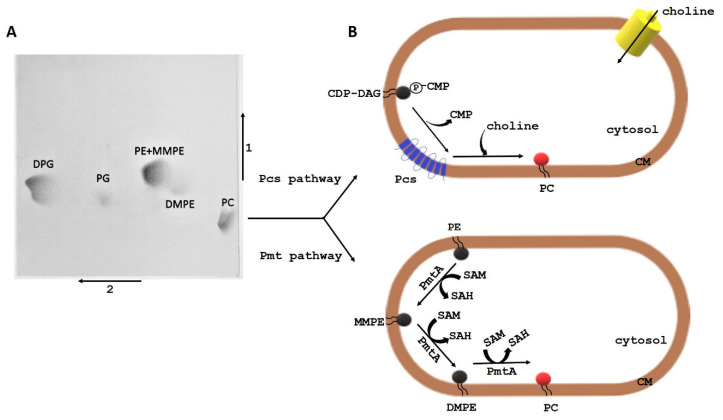
(**A**) Two-dimensional thin-layer chromatogram of phospholipids from *Legionella gormanii*. PC—phosphatidylcholine, DMPE—phosphatidyl-*N*,*N*-dimethylethanolamine, PE—phosphatidylethanolamine, MMPE—phosphatidyl-*N*-monomethylethanolamine, PG—phosphatidylglycerol, and DPG—diphosphatidylglycerol (cardiolipin). Solvent system: (1) first dimension—chloroform/methanol/water (14:6:1, *v*/*v*/*v*) and (2) second dimension—chloroform/methanol/glacial acetic acid (13:5:2, *v*/*v*/*v*). (**B**) PC biosynthesis pathways in *L. pneumophila*. CDP, diacylglycerol; Pcs, phosphatidylcholine synthase; PmtA, phospholipid *N*-methyltransferase; SAM, *S*-adenosylmethionine; SAH, *S*-adenosylhomocysteine; and CM, cytoplasmic membrane.

The following phospholipid classes have been identified in the membranes of *L. pneumophila*, *L. lytica*, *L. bozemanae*, *L. dumoffii*, *L. anisa*, *L. gormanii*, and *L. longbeachae*: phosphatidylcholine (PC), PE, CL, and PG [21,22,23,24,25] (Figure 2A). PC and PE are the major *Legionella* phospholipids. The relative amount of PC in the different species ranges from 30% to 50% of all PLs. PE dominates in the lipids of *L. anisa*, *L. gormanii*, and *L. longbeachae* [25]. The distribution of phospholipids in the particular layers of the *L. pneumophila* bacterial envelope is as follows: the outer layer of the cytoplasmic membrane (CM-1) contains ca. 43% PC, 45% PE, and 12% CL, while the inner layer (CM-2) has 35% PC, 42% PE, 14% CL, and 8% PG. PE dominates in the outer (OM-1) and inner layers (OM-2) (ca. 50% in each layer), whereas PC constitutes 27% and 33%, respectively, CL represents 10% and 6%, respectively, and PG accounts for ca. 13% [26].

### 2.2. Fatty Acid Composition of Legionella spp.

The major fatty acyl residues in *Escherichia coli* phospholipids are palmitic, stearic, *cis*-vaccenic, and cyclopropane-containing lactobacillic acids [19]. An analysis of the fatty acid (FA) composition of *Legionella* spp. showed the presence of cyclopropyl heptadecanoic acid and a high content of branched (*iso* and *anteiso*) FAs (40–90%). The cellular FAs are dominated by 14-methylpentadecanoic (i16:0), 12-methyltetradecanoic (a15:0), and 14-methylhexadecanoic (a17:0) acids. On the basis of the differences in the relative amounts of 14-methylpentadecanoic (il6:0), hexadecenoic (16:0), and 12-methyltetradecanoic (a15:0) acids, Lambert and Moss divided the investigated *Legionella* species into three major groups (16C, A15, and A15/16C). The 16C group includes species with a high content of i16:0 or 16:1 (with one unsaturated bond) or both fatty acids. The A15 group includes species with a high content of a15:0 at concentrations approximately twice that of i16:0. The third group comprises species with a15:0 and i16:0 as major fatty acids in approximately equal amounts [27]. An analysis of the fatty acid contents in the individual *L. dumoffii* lipid classes showed that PC and PE contained high amounts of a15:0 and cyclopropyl 17:0 fatty acids. The PC fraction was also characterised by the presence of unbranched hexadecenoic acid, while PE had a17:0 fatty acid. Similar to PC and PE, CL contained significant amounts of a15:0 and branched i16:0. Saturated and unbranched hexadecenoic and octadecanoic fatty acids dominated in the PG fraction [28].

Detailed information on the structure of phospholipids, i.e., the distribution of fatty acids in the *sn-1* and *sn-2* positions of the glycerol backbone, was provided by liquid chromatography (LC) coupled with mass spectrometry (MS). Besides components that were common to *L. bozemanae*, *L. lytica*, and *L. dumoffii*, e.g., PC16:0_15:0 and PE15:0_15:0, the species differed in the composition of fatty acids bound with different classes of phospholipids. In the phosphatidylcholine class, PC17:0_15:0 was characteristic for *L. bozemanae*, PC16:0_14:0 and PC18:0_16:1 for *L. lytica*, and PC16:0_17:1 (or cyclopropyl 17:0) for *L. dumoffii*. PE16:1_15:0 dominated in *L. bozemanae*, PE14:0_14:0 and PE15:0_14:0 in *L. lytica*, and PE15:0_15:1 in *L. dumoffii*.

The differences in the composition of fatty acids contained in the phospholipids are an important chemotaxonomic feature and may have practical relevance in the diagnostics of this bacterial group at the species level. Moreover, the aforementioned *Legionella* species synthesised straight-chain acids besides the branched ones, in contrast to *L. pneumophila*, whose phospholipid molecules contained only branched fatty acids [21,22,23,24,25]. The LC-MS/MS method based on the structural analysis of PLs can be applied in *Legionella* diagnostics, but a greater number of species should be examined to create PL databases allowing the identification of *Legionella* species.

*L. pneumophila* change their fatty acid compositions, depending on the growth phase. In the stationary growth phase, the proportion of branched-chain fatty acids rises to over 60%, and the average length of fatty acids in phospholipid molecules decreases, compared to the exponential growth. Since acyl chains attached to phospholipids determine various membrane properties, e.g., fluidity and sensitivity to antibiotics, Verdon et al. showed that changes in the pattern of *L. pneumophila* fatty acids between the growth phases led to an increased tolerance to the antimicrobial peptide warnericin RK [29].

PC is the main phospholipid of *Legionella* bacteria. This zwitterionic phospholipid, characteristic of eukaryotic cells and a narrow group of bacteria, is responsible for many biological functions, including interactions with the host cell.

### 2.3. PC Synthesis Pathways

Among the four experimentally confirmed PC synthesis pathways in bacterial cells, *Legionella* spp. use the phosphatidylethanolamine (PE) methylation (Pmt) pathway and the PC synthase (Pcs) pathway [30] (Figure 2B). In the *N*-methylation pathway, PE is *N*-methylated three times, which leads to the formation of PC. Monomethyl-*N*-phosphatidylethanolamine (MMPE) and dimethyl-*N*,*N*-phosphatidylethanolamine (DMPE) are intermediate compounds in this process. The pathway is catalysed by phospholipid *N*-methyltransferases (PmtA). The enzyme uses *S*-adenosylmethionine (SAM) as the methyl donor, converting it to *S*-adenosylhomocysteine (SAH) [31]. The Pcs pathway is a single-step reaction between choline and CDP-diacylglycerol to form PC [32]. The reaction is catalysed by the phosphatidylcholine synthase (Pcs) present exclusively in bacteria [33].

### 2.4. Genetic Diversity of Legionella pcs and pmtA Genes

As demonstrated by genetic analyses, *Legionella* species contain genes (*pcs* and *pmtA*) encoding enzymes involved in the synthesis of PC via two independent pathways. A comparative analysis of the nucleotide sequences of the *pcs* gene encoding Pcs has shown the high sequence identity of these genes among members of the *Legionellaceae* family (in a range from 64% to 98%). *L. bozemanae*, *L. anisa*, *L. gormanii*, *L. parisiensis*, and *L. tusconensis* were found to form one clade on the phylogenetic tree, which indicates that they are closely related. The three strains of *L. pneumophila*, *L. fallonii*, and *L. micdadei* exhibit a considerable phylogenetic distance from all non-*L. pneumophila* species [25]. The *pcs* genes in all analysed *Legionella* spp. exhibit a high conservation degree, although the genome of these bacteria is highly heterogeneous, with over 60% species-specific genes [34]. *Legionella* Pcs proteins encoded by *pcs* genes have a similar length of 254 amino acids the exceptions are *L. pneumophila* (255 aa) and *L. longbeachae* (253 aa). These proteins contain a highly conserved 27-aa-long motif DGX_2_ARX_8_PX_3_GX_3_DX_3_D, and the amino acid motif is shared with the CDP-alcohol phosphotransferase superfamily [35]. The Pcs proteins are highly hydrophobic. They contain up to eight transmembrane helices with *N*- and *C*-termini in the cytoplasm. Their *N*-terminal region has a domain responsible for enzymatic activity.

In comparison with the *pcs* genes, *Legionella pmtA* genes encoding PmtA enzymes exhibit higher sequence diversity and lower sequence identity to each other. As revealed by comparative sequence studies of the *pmtA* genes, *L*. *longbeachae* is closely related to *L. sainthelensi* (92% sequence identity), *L. cincinnatiens* (89%), *L. santicrucis* (91%), and *L. gratiana* (87%). However, no *pmtA* sequences for *L. drancourtii*, *L. gormanii*, *L. dumoffii*, and *L. micdadei* have been identified with the use of degenerative primer pairs homologous to *Legionella*, *Rhodobacter*, and *Rhizobium* bacteria. PmtA sequences have been found to diverge substantially among *Legionella* spp. at the amino acid level. The PmtA protein of *L. pneumophila* exhibited only 65–70% aa sequence identity to the proteins from other *Legionella*. The *pmtA* genes encode small 208–218-aa-long cytosolic proteins. The catalytic domain of these proteins includes a 9-aa-long motif: V/ILE/DXGXGXG. It is predicted to bind the methyl donor S-adenosylmethionine (SAM) and is characteristic for methyltransferases [36]. At least two PmtA families have been described in bacterial cells. One family is similar to the PmtA of *Rhodobacter sphaeroides* (Rs-PmtA), whereas the other type is similar to the PmtA of *Ensifer meliloti* (Sm-PmtA) [37]. The *Legionella* PmtA enzymes exhibit homology to the *Rhodobacter* Pmt-type enzyme, which, in turn, is homologous to UbiE (ubiquinone/menaquinone biosynthesis methyltransferase). The single PmtA enzyme in *Legionella* spp. catalyses successive PE *N*-methylation via the MMPE and DMPE intermediates to form PC. Interestingly, as shown by an analysis of the composition of *L. dumoffii* phospholipids, the bacterium produces methylated derivatives of PE, which provides evidence for the presence of the Pmt pathway in PC synthesis. However, the absence of a *pmtA* homologue in the *L. dumoffii* genome may suggest the presence of a new type of enzyme with PmtA activity, different from Sm-PmtA or Rs-PmtA.

A method for identification and discrimination between *Legionella* species in the set of duplex-PCR was specified based on the *16S rRNA* gene and the *pcs* and *pmtA* genes encoding PC synthase and PmtA, respectively [38].

### 2.5. Utilisation of Exogenous Choline for PC Synthesis by Legionella spp.

The use of exogenous choline by *Legionella* spp. in the Pcs pathway leads to a change in the contents of their individual classes of phospholipids. ^31^P nuclear magnetic resonance (NMR) spectroscopy showed that *L. gormanii* cultured on a choline-supplemented medium synthesised by 21% and 12% higher PC and PE contents, respectively, and by 9% lower CL levels in comparison with bacteria grown on the medium without choline [25]. A comparative analysis of the contents of each PL class revealed that *L. dumoffii* grown on choline synthesised by 12% higher PC levels and by 12% lower PE amounts than bacteria cultured without the addition of choline [28]. *L. pneumophila* grown on the medium with choline produced by 6% and 3% higher quantities of PC and PE, respectively, and by 9% lower amounts of CL compared with bacteria grown on the medium without choline. However, the contents of individual classes of PLs in extracts from *L. anisa* and *L. longbeachae* cells were unchanged, irrespective of the culture conditions [25]. *L. bozemanae* and *L. dumoffii* grown on BCYE medium supplemented with *N*-nonadeuterotrimethyl-choline chloride synthesised labelled PC species. The occurrence of labelled PC such as d_9_-PC16:0_15:0, d_9_-PC15:0/15:0, d_9_-PC17:0_15:0, d_9_-PC16:1_15:0, d_9_-PC17:0_17:0 cyclopropyl, and d_9_-PC15:1_15:0 in *L. bozemanae* phospholipids, as well as d_9_-PC14:0_16:0, d_9_-PC16:0_17:1 (or 17:0 cyclopropyl), and d_9_-PC15:0_15:0 in *L. dumoffii* phospholipids, indicated the utilisation of exogenous choline by these bacteria. An analysis of the distribution of labelled PC in the outer (OM) and inner (IM) membranes in *L. dumoffii* revealed that phosphatidylcholines derived from the Pcs pathway are located in both membranes. The selective MRM (multiple reaction monitoring) method showed greater amounts of PC in the inner membrane. A comparison of ion intensities in the mass spectra (Matrix-Assisted Laser Desorption/Ionization/Time-of-Flight, MALDI-TOF) of PC (derived via the Pcs pathway) with unlabelled PC (derived via the Pmt pathway) indicated that both PC synthesis pathways operate simultaneously in *L. bozemanae* and *L. dumoffii* cells. The Pcs pathway was found to be a dominant, and probably more energy-efficient pathway [23,24,28]. The *L. pneumophila pmtA* mutant, in which the Pcs pathway depends on extracellular choline, synthesised almost all the PC, while the *pcs* mutant with the functioning Pmt pathway produced only 6% of PC [39].

The utilisation of extracellular choline was found to influence the composition of *L. gormanii* fatty acids. The bacterium cultured on choline nonsupplemented medium synthesised more a15:0 fatty acid and larger amounts of n16:0, 16:1, and cyclopropyl 17:0 fatty acids when grown on the medium with choline [25]. However, the addition of choline to the growth medium did not induce qualitative and quantitative changes in the fatty acid profiles in *L. pneumophila*, *L. anisa*, and *L. longbeachae* cells [25].

Exogenous choline induced changes not only in lipids and the fatty acid composition but, also, in proteins, as shown by FTIR (Fourier-Transform Infrared with Attenuated Total Reflection) spectroscopy. After choline supplementation, higher concentrations of proteins represented by the amide I and amide II bands in *L. anisa*, *L. gormanii*, and *L. longbeachae* were detected [25].

### 2.6. Biological Importance of Legionella PC

*Legionella* rods belong to the narrow group of bacteria synthesizing PC, which, in addition to membrane formation, plays important roles in bacterial–host interactions ranging from symbiosis to pathogenesis [40]. The presence of PC in cell membranes is specific to pneumonia-causing bacteria. The lung surfactant covering the small airways, bronchioles, and alveolar surface is composed of approximately 10% of protein and 90% of lipids, with approximately 80% of the lung surfactant lipids representing PC. *P. aeruginosa* utilises lung surfactant PC as a primary carbon and energy source, which facilitates high-cell density replication during lung infection [41]. In cystic fibrosis patients, in whom *P. aeruginosa* is a common cause of pulmonary infections, choline, i.e., a soluble product of PC and sphingomyelin hydrolysis, is a putative osmoprotectant in the high osmolarity environment of the lung [42]. The PC synthesis pathway (Pcs) can play the role of an environmental sensor, since the biosynthetic precursors present in lung tissue may exert an effect on *L. pneumophila* virulence via PC incorporation into the bacterial cell envelope [39]. Rapid PC synthesis might be required in *Legionella* spp. to quickly adjust their membrane physiology to new environmental conditions [43]. A PC-deficient *L. pneumophila* strain showed attenuated binding to macrophages and a defect in the functioning of the IV secretion system, which is essential for effector transport and formation of the intracellular replication niche in alveolar macrophages. The lack of PC in *L*. *pneumophila* membranes resulted in reduced cytotoxicity and impaired capability of the intracellular proliferation of these bacteria, which indicates an important function of this phospholipid in the interaction with the host cell [39]. The utilisation of extracellular choline and incorporation of PC into *Legionella* membranes lead to changes in the compositions (lipids and proteins) of cell membranes, which influences their physicochemical properties and interactions with external factors (other cells and antimicrobial peptides). Atomic force microscopy measurements revealed changes in the nanomechanical properties of the cell surface of *L. dumoffii* grown with extracellular choline, i.e., a two-fold increase in the Derjaguin–Muller–Toporov (DMT) modulus reflecting elasticity. A considerably more effective binding of apolipophorin III (apoLp-III, an insect homologue of human apolipoprotein E) to bilayers composed of PLs extracted from choline-grown *L. dumoffii*, thus enriched with PC, was observed [28]. This is in line with the study conducted by Zhang, which demonstrated a stronger binding of apoLp-III to PC compared to PE, indicating an important role of cell membrane PC in the interaction with apoLp-III [44]. Interestingly, the elasticity of the cell surface of choline-cultured *L. dumoffii* treated with apoLp-III did not change in contrast to the six-fold increase in the cell envelope elasticity of these bacteria grown without choline after incubation with apoLp-III [45]. Another possible explanation for the stronger binding of apoLp-III to lipids isolated from *L. dumoffii* grown on choline is the change in the ratio between the PC and PE contents in these bacteria and the resulting alterations in the membrane architecture. PC formed a bilayer structure, whereas PE made the membrane more rigid. The 12% reduction in the PE content in membranes of choline-cultured *L. dumoffii* may be the cause of the less-tight membrane packing and easier apoLp-III binding. Similar to apoLp-III, defensin isolated from *Galleria mellonella* haemolymph was over three times more active against *L. dumoffii* when the bacterial cells were cultured in the presence of exogenous choline [46]. However, the differences in the morphology of the cells visualised with the use of the electron microscope, resulting from the action of apoLp-III and defensin, indicate a different mechanism of their interaction with *L. dumoffii*.

PC may serve as a specific recognition molecule. *L. pneumophila* PC is required for the binding of these bacteria to macrophages via the platelet-activating factor receptor (PAF receptor), and the efficiency of this process depends on the content of PC. The adhesion of *L. pneumophila* to macrophages was successfully blocked by a PAF receptor antagonist [39]. Chemically, PAF is 1-*O*-alkyl-2-acetyl-*sn*-glycerol-3-phosphocholine, and, due to this structural similarity, *L. pneumophila* PC can mimic PAF and, by binding to its receptor, increase the chance of bacterial uptake by macrophages. *L. dumoffii* cells with increased PC contents underwent internalisation by macrophages of the THP-1 (the human monocytic leukemia cells) line more readily than bacteria with a reduced content of this phospholipid [24]. *Legionella* PC may influence the modulation of the inflammatory functions of macrophages measured by the level of proinflammatory cytokines (tumour necrosis factor, TNF-α and interleukin-6, IL-6). These cytokines are involved in the protective immune response controlling the early stages of *L. pneumophila* infection. The treatment of animals with either TNF-α or IL-6 resulted in a significant reduction in mortality caused by *L. pneumophila* infection.

Human phagocytic THP-1 cells, which differentiate into macrophages after treatment with phorbol 12-myristate 13 acetate (PMA), have been investigated. PMA-triggered THP-1 differentiation causes a rearrangement of the macrophage-specific kinome towards a more proinflammatory phenotype [47]. *L. dumoffii* grown on a choline-supplemented medium at a dose of 10 and 100 MOI (multiplication of infection) induced over a three-fold lower level of TNF-α in THP-1 macrophages than bacteria cultured without the addition of choline. Similarly, the inner membrane of *L. dumoffii* isolated from bacteria grown on the choline-supplemented medium was a weaker TNF-α inducer than the membrane of bacteria grown without choline. Statistically significant results were obtained in the presence of 100 ng/mL of the inner membrane. In turn, the same dose of the outer membrane of bacteria cultured with choline was a better cytokine inducer than the outer membrane of bacteria cultured without choline supplementation [24]. *L. anisa*, *L. longbeachae*, *L. gormanii*, and *L. pneumophila* grown on a medium with choline caused much lower TNF-α production, with the highest decrease in the TNF-α level for *L. longbeachae*, compared to those bacteria grown without the choline addition [25]. The supplementation of growth medium with choline was also the cause of a decrease in IL-6 production after the incubation of macrophages with *L. pneumophila*, *L. longbeachae*, and *L. anisa*, with the highest decrease by 52% in the level of IL-6 for *L. anisa* [25]. The exact mechanism of the weaker induction of proinflammatory cytokines by *Legionella* spp. grown on the choline-supplemented medium compared to those cultured without exogenous choline requires elucidation. A still open question is whether there are different mechanisms responsible for the attenuated production of proinflammatory cytokines, since there are differences among the various *Legionella* spp. in the PC structure and content, depending on culture conditions.

Changes in the contents and structures of membrane PCs may affect the contents and activity of other cell components (e.g., other classes of phospholipids and proteins), and the observed decrease in the proinflammatory cytokine production may result from the effect of various factors on the macrophages. PC synthesis in the Pcs pathway can lead to a change in the proportion between zwitterionic (PC and, PE) and anionic (CL and PG) lipids, resulting in an imbalance in the electrostatic charge of the cell membrane. Since such a balance is required for many integral membrane proteins to adopt the correct topology in the cell membrane, changes in mutual relations between PLs can lead to modifications in the structure of the transmembrane α-helices of membrane proteins, altering the packing of these helices [48,49]. All of this induces surface changes in bacterial ligands and may lead to altered interactions with macrophages.

The less potent proinflammatory cytokine induction under the influence of bacteria grown on a choline-supplemented medium, in comparison with that induced by bacteria grown without a choline addition, may be one of the ways for bacterial evasion of the host immune system. To survive and multiply in host cells, *Legionella* rods have developed sophisticated adaptation mechanisms such as the ability to utilise extracellular choline sources. Selective blocking of phosphatidylcholine synthase can become a target in the specific therapeutic effect supporting the treatment of Legionnaires’ disease. *Legionella* spp. are able to synthesise PC via two independent pathways, and potential drugs that block the activity of phosphatidylcholine synthase may contribute to the attenuation of the virulence trait.

## 3. Structure and Significance of *Legionella* Lipopolysaccharide

### 3.1. Chemical Structure of Legionella LPS

Lipopolysaccharide is a glycolipid composed of three covalently linked regions: lipid A, a core oligosaccharide featuring an outer and inner region, and an O-specific chain. Although LPS isolated from various *Legionella* species share common features in their basic architecture, the LPS of *L. pneumophila* differs significantly in the chemical structure and biological significance of the individual parts. The O-specific chain of the *L. pneumophila* Philadelphia strain consists of a homopolymer of α-(2-4)-linked 5-acetamidino-7-acetamido-8-O-acetyl-3,5,7,9-tetradeoxy-l-glycero-d-galacto-non-2-ulosonic acid [50]. This unusual sugar, termed legionaminic acid, is highly hydrophobic due to the presence of acetyl and acetimidoyl groups. The O-specific chain is bound to a seven-sugar outer core composed of rhamnose (Rha), mannose (Man), acetylquinovosamine (QuiNAc), and acetylglucosamine (GlcNAc) in the molar ratio of 2.1:1.1:1:1.4 [51]. The presence of *N*-acetyl groups (QuiNAc and GlcNAc); methyl groups of 6-deoxy sugars (Rha and QuiNAc); and *O*-acetyl groups at C2 of Rha, C4 of QuiNAc, and C3 of GlcNAc makes the outer core highly hydrophobic. The hydrophilic inner core consists of two molecules of 3-deoxy-D-manno-2-octulosonic acid bound by a 2→4 ketosidic linkage and one molecule of D-mannose connected to the C8 of Kdo present in the inner core [52]. A characteristic feature of the inner core of LPS from *L. pneumophila* is the absence of heptoses and phosphate residues. The carbohydrate backbone of *L. pneumophila* lipid A consists of a bisphosphorylated disaccharide of the 2,3 diamino-2,3-dideoxy-D-glucose (GlcN3N) amide linked with 3-hydroxy and 2,3-dihydroxy fatty acids [53,54]. The hydroxyl groups of the fatty acids linked directly to the sugar backbone are acylated by straight, branched (*iso* and *anteiso*), and long-chain fatty acids. Lipid A of *L. pneumophila* contains eight nonhydroxylated acids from 14 to 20 carbon atoms and five long-chain fatty acids (28:0(27-OH), 28:0(27-oxo), 30:0(29-oxo), 27-dioic, and 29-dioic) [55].

The high diversity among *Legionella* spp. is also expressed in the structures of the lipopolysaccharides, including the conserved region of lipid A. The lipid A moiety of LPS from most *Legionella* spp. comprises disaccharide GlcN3N. However, a mixed disaccharide backbone containing GlcN3N and glucosamine was found in *L. israelensis* and *L. bozemanae* [56,57]. An unusual feature of lipid A from *Legionella* spp. is the high content of non-hydroxy fatty acids (ester-linked) and long-chain (-ω)-oxo, (-ω)-hydroxy, and (ω)-dioic fatty acids (ester linked) [58]. 27-oxo-octacosanoic acid is generally present in all *Legionella* species studied; therefore, it serves as a useful marker of this bacterial group. An analysis of the sugar components of LPS from different *Legionella* species showed that all structurally characterised lipopolysaccharides displayed high contents of d-mannose and d-glucosamine. In addition, *Legionella* LPS contain species-specific sugars (3 amino-3, 6-dideoxy-mannose in *L. israelensis*, galacturonic acid and galactosamine in *L. hackeliae*, quinovose in *L. feeleii*, and a rare sugar yersinose in *L. micdadei* and *L. maceachernii*) [59,60]. Unlike *L. pneumophila*, LPS isolated from several other species, e.g., *L. feeleii*, *L. jordanis*, *L. erythra*, *L. bozemanae*, *L. oakridensis*, and *L. micdadei* contain d- and l-glycero-d-manno-heptose [56,60,61].

The chemical composition and structure of *Legionella* LPS, especially the presence of various decorative substituents, exerts a strong influence on its biological activity in all stages of host cell infection.

### 3.2. Biological Significance of Legionella LPS

*L. pneumophila* LPS is the main antigen recognised by antibodies contained in the serum of patients and the thermostable antigen excreted in urine [62]. The urinary antigen test is the most commonly used method for the diagnosis of Legionnaires’ disease [63]. The use of monoclonal antibodies (mAb) of the Dresden panel, recognising epitopes located in the highly heterogeneous O-specific chain of *L. pneumophila* LPS, facilitated distinguishing 15 serogroups and nine subgroups within serogroup 1 [64]. The two antibodies (mAb3/1 and mAb8/5) that recognise the virulent strains responsible for the majority of laboratory-confirmed cases of Legionnaires’ disease turned out to be valuable in epidemiological studies and for clinical purposes. The characterisation of the LPS biosynthesis loci of *L. pneumophila* serogroup 1 strains revealed two major regions: a specific 18-kb region and a conserved 15-kb region containing genes found in serogroup 1 and non-serogroup 1 strains. The most variable region is involved in O-antigen modification [65]. Antibody mAb3/1 recognises an epitope associated with the 8-O-acetyl group in legionaminic acid. The O-acetyl-transferase enzyme encoded by the *lag-1* gene is responsible for the transfer of the O-acetyl group to legionaminic acid [66]. Studies carried out in Europe and Asia have shown that the *lag-1* gene was harboured by a significantly higher number of clinical isolates of *L. pneumophila* sg1 compared with environmental isolates [67,68].

The LPS of *L. pneumophila* is highly hydrophobic due to the presence of deoxy groups and *N*- and *O*-acyl substituents in legionaminic acid, but the highest degree of hydrophobicity is exhibited by *lag-1* strains producing 8-*O*-acetyl groups, which most likely contributes to the transmissibility of these bacteria in aerosols and adhesion to host cells. A TF3/1 mutant in the *lag-1* gene defective in the synthesis of 8-*O*-acetyl substituents, not recognised by the mAb3/1 antibody, adhered less strongly to the macrophages of the THP-1 line and to *A. castellanii* cells, compared to the wild-type strain [69]. This mutant also failed to produce high-molecular-weight long-chain O-polysaccharide [70]. Comparative studies of the kinetics of interactions between the host cell and the *L. pneumophila* wild-type strain or the mutant showed a higher efficiency of binding to the amoeba surface in bacteria with the full-length O-chain and 8-O-acetyl groups. However, both strains multiplied inside the host cells successfully, irrespective of the differences in the length and structure of the polysaccharide part of LPS. Previous studies also showed that *L. pneumophila* strains lacking 8-*O*-acetyl substituents were as effective in infecting amoebae and macrophages as strains that expressed this LPS motif [70]. Thus, the LPS of *L. pneumophila* plays a critical role in the early stages of infection, anchoring the bacteria to the host cell’s membrane. Disturbances in the synthesis of the polysaccharide region of *L. pneumophila* LPS exert an effect on the composition and structure of phospholipids and proteins. The TF3/1 mutant showed differences in the structures of the PC and PG species, compared to the wild-type strain. Moreover, the mutant strain was synthesised by 11 mol% lower amounts of branched fatty acids and approximately two-fold higher amounts of long-chain fatty acid (20:0) than the wild-type strain. The changes in the surface components determined the cell surface topography of these bacteria and their nanomechanical properties. The TF3/1 mutant had grooves on the cell surface and did not produce as many outer membrane vesicles (OMV) as the wild-type strain [69]. Spherical bilayers consisting of LPS, phospholipids, outer membrane proteins, and periplasmic components are naturally secreted from the cell envelope of *L. pneumophila*. OMVs play a significant role in the pathogenesis of these bacteria, simultaneously delivering multiple virulence factors to host cells and tissues [71]. The release of the vesicles from *L. pneumophila* is developmentally regulated, i.e., the vesicles are connected to the cell wall but shed LPS in the replicative phase and are profusely released in the transmissive phase [72]. One of the functions of *L. pneumophila* OMVs is the inhibition of phagosome–lysosome fusion during the intracellular infection of macrophages. This capability was correlated with growth phase-dependent modifications of the composition of glycoconjugates contained in the vesicles. The LPS of the bacteria in the transmissive phase of growth is deacetylated and elongated to a form that effectively blocks the fusion between phagosomes and lysosomes and, thus, independently of the effectors of the IV secretion system, inhibits the maturation of macrophages [73]. In turn, during the replicative phase, the degree of acetylation of the LPS O-chain increases, which probably contributes to the increased tolerance to the hostile conditions of the intracellular environment of macrophages [73]. Seeger et al. showed that LPS fractions below 300 kDa, not associated with OMV, significantly delayed phagolysosomal maturation one hour after phagocytosis, regardless of the bacterial growth phase [74].

LPS expressed by *L. pneumophila* in the transmissive phase binds to a sialic acid-specific lectin more strongly than LPS from bacteria in the replicative phase of growth. The ability of the bacteria to bind to this receptor correlates with the effectiveness of macrophage infection [73]. Legionaminic acid of *L. pneumophila* sg1 shares the same d-glycero-d-galacto absolute configuration as 5-acetamido neuraminic acid (Neu5Ac, sialic acid). Neu5Ac, located on the surface of mammalian cells, is involved in cell–cell interactions and the immune response [75]. The molecular mimicry of eukaryotic cell macromolecules is one of the strategies used by *Legionella* bacteria to colonise host cells. The structural similarity of legionaminic and neuraminic acid may be an example of mimicry to the host cell used not only in the adhesion process but, also, in modulation of the immune response to infection. The LPS of *L. pneumophila* is less toxic and less potent in its ability to induce proinflammatory cytokines (IL-1β, IL-6, IL-8, and TNF-α) from Mono Mac 6 cells, compared to the highly pyrogenic LPS from *Enterobacteriaceae* members [76]. The bioactive centre of LPS is lipid A, whose toxicity is directly influenced by the length and number of groups of the fatty acids attached to its glycosidic backbone. The presence of fatty acid chains twice the length of the corresponding chains found in the majority of toxic lipids A containing C12, C12OH, C14, and C14OH account for the low endotoxic activity of *L. pneumophila* LPS due to a failure to interact with receptor CD14 (a glycosylphosphatidylinositol-anchored protein) and with its soluble form [76]. Host pattern recognition receptors such as Toll-like receptors (TLRs) are involved in the process of recognition of LPS. TLR4 functions as a sensor of LPS on the OM in Gram-negative bacteria, promptly inducing the production of antibacterial cytokines. The lipid A region of *L. pneumophila* LPS was a weak TLR4 agonist [77]. Additionally, macrophages of TLR4-deficient mice infected by *L. pneumophila* were not defective in the production of cytokines, and the rate of bacterial clearance from the lungs of these mice was similar to that in wild-type mice [78,79]. However, the TLR2-deficient mice were more sensitive to *L. pneumophila* than TLR2-sufficient mice [79,80]. These results indicated that LPS from *L. pneumophila* was recognised by TLR2, which is a typical receptor for peptidoglycan [77]. This unusual detection pattern of switching TLR4 to TLR2 is related to the presence of substituent (a ketone group at C27) or branch on the penultimate carbon of different fatty acids of *L. pneumophila* lipid A [77]. Upon recognition by TLR2, LPS triggers signalling pathways controlled by the MyD88 adaptor protein, which results in the production of inflammatory cytokines and subsequent clearance of *L. pneumophila* from the lungs [81]. The level of inflammatory cytokine production was reduced in TLR2 and MyD88 gene knockdown macrophages of the U937 cell line infected by *L. pneumophila* [82]. Additionally, human macrophages induced by *L. pneumophila* OMVs synthesised IL-8 relying on TLR2-dependent signalling pathways [71].

The structure of lipid A composed of GlcN3N and long-chain fatty acids also provides a protective mechanism against the degradation of lipid A/LPS by amidases and/or esterases of amoebae during the intracellular growth of *L. pneumophila* [54].

*L. pneumophila* LPS is subject to phase variations correlated with the attenuation of virulence traits such as the ability to multiply within macrophage-like HL60 cells or *A. castellanii* [83]. The molecular mechanism responsible for this variability consists in the chromosomal insertion and excision of an unstable 30-kb genetic element, which does not harbour genes related to LPS biosynthesis [84]. Phase variation in the RC1 strain of the *L. pneumophila* sg 1 subgroup OLDA influences the O-specific chain structure and the fatty acid profile of lipid A. The phase-variant strain 811 with the 29-kb element excised from the chromosome is devoid of *N*-methylation in legionaminic acid and contains shorter 3-hydroxy (16:0 and 18:0) fatty acids in lipid A [85,86].

*L. pneumophila* strain PtVFX/2014, associated with the first evidence of person-to-person transmission, i.e., a strain that was able to overcome the transmission barrier of human innate immunity, carried eight horizontally transferred regions encompassing genes involved in, e.g., LPS biosynthesis [87]. Epidemiological studies showed that the genes of the LPS cluster determining sg 1 of *L. pneumophila* were present in highly diverse genomic backbones of the strains responsible for the largest outbreaks of Legionnaires’ disease described so far and that it probably constitutes a major determinant of human disease itself [87].

On the one hand, the LPS composed of three distinct regions in terms of structure and biological properties is an important factor in the virulence of *Legionella* bacteria, participating in complex mechanisms of the induction of lesions. On the other hand, LPS is a ligand recognised by proteins involved in the response to infection. During the infection of macrophages with *L. pneumophila*, host guanylate binding proteins (GBPs), triggered by cytoplasmic LPS derived from the bacteria, induce caspase-11-dependent pyroptosis [88,89]. The LPS of *L. pneumophila* is a ligand for collectins, which play an important role in the innate immunity of the lung. After the direct binding of LPS, hydrophilic proteins A and D promote the localisation of *L. pneumophila* in the acidic environment of the lysosomes, thus attenuating the intracellular multiplication of the phagocytosed bacteria [90]. Additionally, human apolipoprotein E binds to *L. pneumophila* LPS, which results in disturbances in the normal structure of the bacterial surface, and these changes may lead to impaired penetration of the host cells [91]. Two molecules of apoLp-III bind to a single micelle of *L. dumoffii* LPS formed from 12 to 29 monomeric LPS molecules, pointing to new strategies for anti-*Legionella* therapies [45].

*Legionella* spp. are important aetiological agents of pneumonia. They are responsible for 2–8% of community-acquired pneumonia cases [92]. A review of 46 CAP (community acquired pneumonia) studies from European countries has indicated that *Legionella* spp. are particularly frequent among patients who require admission to an intensive care unit (ICU) [93]. In the USA, the reported cases of Legionnaires’ disease increased from 2.301 in 2005 to 7.104 in 2018. In Europe, the number of known cases of Legionnaires’ disease has almost doubled (from 1.3/100,000 people in 2014 to 2.3/100,000 people in 2018) [94]. The increase in cases may be related to the changing environmental conditions, which favour the growth of *Legionella* bacteria, as well as the increasing number of people susceptible to infection, such as the elderly and immunocompromised subjects. The closure of public buildings related to the SARS-CoV-2 pandemic has led to the long-term stagnation of water in installations and created optimal conditions for intensive multiplication of the bacteria, which substantially increased the risk of *Legionella* infections [95]. *L. pneumophila* produces a variety of cytosolic and cell envelope-associated lipids with a unique structure that plays an important role in its physiology and promotes bacterial adhesion and adaptation to the host’s intracellular environment.

Although more than 60 different *Legionella* species are known, with approximately half of the number of species isolated from clinical specimens, the complete structure of LPS is only known in *L. pneumophila*. The analysis of the genomes of various *Legionella* species has shown that these bacteria contain genes whose products are involved in the synthesis of lipids characteristic for eukaryotic cells, such as PC, or, e.g., hopanoids in *L. falonii* [96]. Lipid components, especially the unique ones, can serve as important biomarkers used in diagnostics and in taxonomic studies of this bacterial group. Bacterial lipid membranes are targets for the development of new antimicrobial drugs and a promising object of research in the fight against resistant bacteria [97]. Therefore, the elucidation of the complex lipid structure of the cell envelope of various *Legionella* spp. using advanced lipidomic technologies may be important for the development of new strategies for the prevention and treatment of *Legionella* infections.

## Data Availability

Not applicable.
